# Quantifying Deviations from Gaussianity with Application to Flight Delay Distributions

**DOI:** 10.3390/e27040354

**Published:** 2025-03-28

**Authors:** Felipe Olivares, Massimiliano Zanin

**Affiliations:** Instituto de Física Interdisciplinar y Sistemas Complejos (CSIC–UIB), Campus UIB, 07122 Palma, Spain; massimiliano.zanin@gmail.com

**Keywords:** ordinal patterns, Jensen–Shannon divergence, air traffic management, flight delays, non-Gaussian distributions, stable distributions

## Abstract

We propose a novel approach for quantifying deviations from Gaussianity by leveraging the Jensen–Shannon distance. Using stable distributions as a flexible framework, we analyze the effects of skewness and heavy tails in synthetic sequences. We employ phase-randomized surrogates as Gaussian references to systematically evaluate the statistical distance between this reference and stable distributions. Our methodology is validated using real flight delay datasets from major airports in Europe and the United States, revealing significant deviations from Gaussianity, particularly at high-traffic airports. These results highlight systematic air traffic management strategy differences between the two geographic regions.

## 1. Introduction

Several methodologies focus on estimating entropic quantifiers for characterizing the dynamical behavior of a system based on time series analysis [1]. In particular, structural entropies typically depend on a predefined probability distribution function, which transforms the time series from the time domain into the frequency/time–frequency domain or a symbolic representation. However, selecting an appropriate methodology—one capable of accurately capturing the intrinsic dynamical properties of the system—represents an ongoing challenge. This difficulty arises from the specific characteristics of the time series, such as its stationarity, length, and level of noise contamination [2]. Notable techniques include Fourier transformation [3], wavelet transform [4], procedures based on amplitude statistics [5], binary symbolic dynamics [6], and ordinal patterns [7].

Ordinal patterns provide a symbolic representation of a given time series by focusing solely on the relative ordering of its values [7]. In this manner, the sequence of symbols emerges organically from the time series, eliminating the need for model-based assumptions. Importantly, ordinal patterns account for the temporal information within the time series, assuming only a weak stationary condition [7]. Thanks to these attributes, they have been widely applied to several scientific fields, including neuronal activity, epileptic events, self-similarity in nature, communications systems, sport science, finance, air transport, etc. (see Refs. [8,9,10] and references therein).

One major drawback of using ordinal patterns is that they discard amplitude information [7]. This omission can lead to a loss of critical information, especially in systems where the absolute magnitude of fluctuations is important [11], such as biomedical signals [12] and extreme event detection [13]. To overcome this limitation, several improvements have been proposed [12,13,14,15]. A comparative study evaluating the classification performance of these approaches can be found in [11]. Nevertheless, all of these approaches primarily focus on signal segmentation and spike detection [11]. Consequently, a quantitatively ordinal characterization of the corresponding amplitude distribution has not been thoroughly explored.

In this study, we demonstrate that analyzing the ordinal pattern distribution of the walk of a time series, rather than its fluctuations, allows for a comprehensive characterization of its amplitude probability distribution. By leveraging the Jensen–Shannon distance permutation [16], a recently introduced metric that quantifies differences between two ordinal pattern distributions, we conduct a quantitative characterization of the ordinal dissimilarity between non-Gaussian and Gaussian distributions by treating phase-randomized surrogates of the former as reference sequences. We employ the family of stable distributions, which enable a quantitative analysis of heavy tails and asymmetry (skewness) [17]. Notably, this distribution family extends the Central Limit Theorem to skewed heavy-tailed processes, providing a more accurate representation of real-world phenomena, such as commodity prices [18]. This property makes stable distributions particularly suitable for analyzing complex systems where Gaussian assumptions fail, including air traffic delays [19,20,21,22]. Understanding these deviations is crucial, as many forecasting and risk assessment models rely on normality assumptions [23,24]. Consequently, detecting and quantifying these deviations is essential for improving predictive models [25,26] and enhancing passenger satisfaction [27]. Here, our approach is validated using flight delay datasets from major airports in Europe and the United States (US). The analysis reveals significant deviations from Gaussianity, particularly at high-traffic airports, with systematic differences between European and US air traffic management strategies.

## 2. Ordinal Patterns and the Permutation of Jensen–Shannon Distance

The ordinal pattern probability distribution, introduced by Bandt and Pompe [7], relies on transforming a time series into a symbolic representation by encoding the relative ranking of the values within subsets of length *D* (D∈N, the pattern length). Specifically, given a sequence of values $X\left(t\right)=\left\{x_{t};t=1,\ldots ,M\right\}$, an ordinal pattern is defined by the permutation $\pi_{i}$ of the indices, {0,1,…,D−1}, that sorts the elements in ascending order. By counting the number of times each ordinal pattern $\pi_{i}$ appears in the encoded time series X(t), normalized by the total number of ordinal patterns M−(D−1)τ, the ordinal pattern probability distribution can be computed:

(1) $$p_{i}=\frac{\#(\pi_{i})}{M-(D-1)\tau },\;\;\;i=1,2,\ldots ,D!,$$

where $\#(\pi_{i})$ stands for the cardinality of $\pi_{i}$. Note that the values of X(t) can be mapped into subsets of length *D* of consecutive (τ=1) or non-consecutive (τ>1) values. In their seminal work [7], Bandt and Pompe propose setting τ=1 as a standard choice. However, selecting different values of τ can offer further insights into the characteristic scales of the system’s dynamics, as it effectively represents multiples of the sampling time [28,29,30,31]. The ordinal pattern distribution has been widely employed in the computation of permutation entropy [7], which quantifies disorder based on the diversity of observed ordinal patterns [32].

Beyond entropy-based quantifiers, an important challenge in time series analysis is to measure the dissimilarity between different dynamical systems. To address this, the Jensen–Shannon distance permutation  [16], in short pJSD, has been introduced as a metric that quantifies the divergence [33] between two ordinal pattern distributions $P=\{p_{1},\ldots ,p_{N}\}$ and $Q=\{q_{1},\ldots ,q_{N}\}$ associated with two time series under analysis,

(2) $$D_{\mathrm{JS}}\left(P,Q\right)=S\left(\left(P+Q\right)/2\right)-S\left(P\right)/2-S\left(Q\right)/2,$$

where $S\left(P\right)=\sum_{i=1}^{N}p_{i}\ln p_{i}$ is the classical Shannon entropy. The pJSD is hence obtained by calculating the square root of Equation (2), and its normalized version reads

(3) $$\mathrm{pJSD}\left(P,Q\right)=\sqrt{\frac{D_{JS}\left(P,Q\right)}{\ln2}},$$

which provides a bounded (pJSD ∈[0,1]), symmetric, and interpretable measure of the ordinal distance between two time series. The pJSD provides a quantitative framework for comparing two time series by measuring how their ordinal pattern distributions differ. Furthermore, it has a key advantage, as hypothesis tests regarding the nature of an arbitrary time series can be easily performed by calculating its pJSD to reference time series generated in agreement with a null model [31] or surrogate analysis [34]. Note that time series originating from the same underlying dynamical system are expected to exhibit small pJSD values, asymptotically approaching zero following a power-law behavior with *M* (pJSD $\propto M^{-1/2}$), but never exactly reaching it due to finite-size constraints [16].

## 3. Numerical Analysis

### 3.1. Stable Distributions

**Figure 1:**
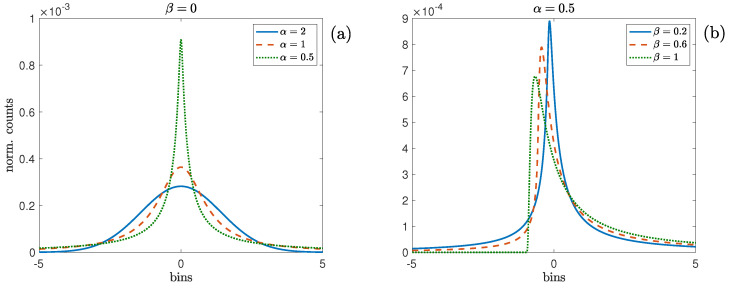
Probability density functions of stable distributions for the (**a**) symmetric cases with β=0 and α=2 (Gaussian), α=1 and α=0.5, and (**b**) skewed cases with α=0.5 and β=0.2, 0.6 and 1. In all cases, δ=1 and γ=0.

A random variable *X* is stable if and only if $X\overset{d}{=}\gamma Z+\delta$ (the symbol $\overset{d}{=}$ means equality in distribution), with γ≠0, δ∈R, and *Z* being a random variable with the characteristic function

(4) $$E\exp\left(itZ\right)=\left\{\begin{array}{cc} \exp\left[-{\left|t\right|}^{\alpha }\left(1-i\beta \mathrm{sign}\left(t\right)\tan\frac{\pi \alpha }{2}\right)\right], & \alpha \neq 1,\\ \exp\left[-\left|t\right|(1+i\beta \frac{2}{\pi }\mathrm{sign}\left(t\right)\log\left|t\right|\right], & \alpha =1, \end{array}\right.$$

with t∈R. The above definition shows that a family of stable distributions (SDs) is defined by four parameters: a stability exponent α∈(0,2], a skewness parameter β∈[−1,1], a scale parameter δ∈(0,∞), and a location parameter γ∈(−∞,∞) [17]. It is important to note that the scale parameter is not the same as the standard deviation (even for Gaussian case α=2), and the location parameter is not typically the mean. The parameters α and β determine the shape of the distribution. On one hand, as α grows, the weights of the tails of the distribution decrease until converging to Gaussianity for α=2, as shown in Figure 1a with β=0, i.e., symmetric case. On the other hand, asymmetry is determined by β, as it increases/decreases from zero, the distribution skews to the right/left. This feature is depicted in Figure 1b with α=0.5 and β>0. It is important to note that as α increases, the effect of β decreases, and for α=2, the skewness of the distribution in independent of β.

### 3.2. Synthetic Data Generation

We consider synthetic uncorrelated time series sampled from this family of stable distributions for different values of α∈[0.5,2] with a step of Δα=0.1 and skewed distributions β∈[−1,1] with a step of Δβ=0.1. For generating the synthetic sequences x(t), we have used the MATLAB R2023b (23.2.0.2365128) function random that follows the parametrization described in Ref. [17]. We have generated one hundred independent realizations of length $M=10^{5}$ data points. Then, the walk of the synthetic data is calculated as $Y_{i}=\sum_{t=1}^{i}\left(x_{t}-\gamma \right)$ with i∈{1,2,3,⋯,M}. Note that the location parameter γ is equal to the mean of the distribution for α>1. For the present analysis, we set γ=0 and δ=1.

### 3.3. Surrogate Analysis

It is our aim here to characterize deviations from Gaussianity as the shape parameters α and β change by measuring the distance between the ordinal distribution of the synthetic data to the one obtained from the phase-randomized surrogate realizations [35], which establishes a Gaussian reference. Nevertheless, these Gaussian references can still yield positive values of the pJSD due to statistical fluctuations. For tackling this, the distances are normalized by defining a Z-score using the average, $\langle pJSD_{FFT}\rangle$, and the standard deviation, $\sigma_{pJSD_{FFT}}$, of the values resulting from the distance between a pair of two phase-randomized realizations from the synthetic data:

(5) $$Z_{pJSD}=\frac{pJSD-\langle pJSD_{FFT}\rangle }{\sigma_{pJSD_{FFT}}}.$$

One hundred independent realizations have been considered. As a result, the normalized distance, $Z_{pJSD}$, quantifies how many standard deviations a given stable distribution is away from the Gaussian reference dataset. The normalized distances are expected to be zero for distributions that coincide with the average obtained by the phase-randomized surrogate set. Conversely, positive values indicate the magnitude (in standard deviations) of the deviation from that average. Normally, for $Z_{pJSD}<3$ (99.7%), the observed distribution is considered Gaussian. We set τ=1, since temporal information is not relevant for our analysis.

**Figure 2:**
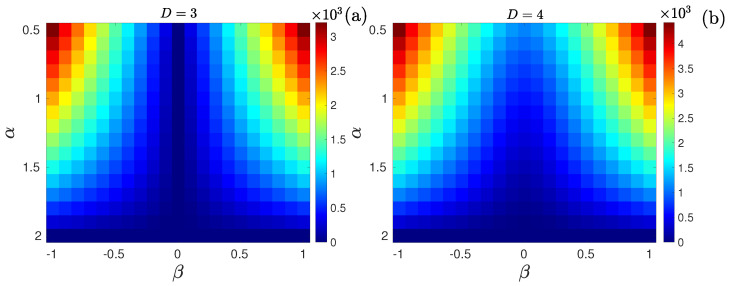
$Z_{pJSD}$ between sequences following stable distribution and their phase-randomized surrogates as a function of the parameter β∈[−1,1] and α∈[0.5,2] with a step of 0.1, for (**a**) D=3 and (**b**) D=4. Synthetic sequences of $M=10^{5}$ data points. The average over 100 independent realizations is reported.

$Z_{pJSD}$ as a function of β and α is shown in Figure 2 for D=3 (a) and D=4 (b). For the symmetric case, β=0, the pattern length D=3 does not detect deviation from Gaussianity as α decreases, i.e., the presence of heavy tails. This is not the case for D=4. For β∈[−1,0)∪(0,1], the $Z_{pJSD}$ correctly quantifies deviations from Gaussianity indistinctly of the sign of the skewness. Naturally, the distance depends on the value of the pattern length since the larger the value of *D*, the more information about the distribution is captured. Finally, we found that for α=2, the distance remains close to zero independently of the pattern length.

**Figure 3:**
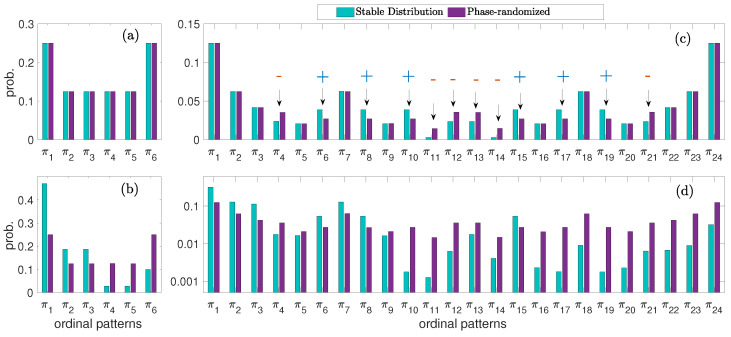
Ordinal pattern probabilities obtained from sample data following a stable distribution with α=0.5 and (**a**) β=0 and D=3, (**b**) β=1 and D=3, (**c**) β=0 and D=4 and (**d**) β=1 and D=4. Average over 100 independent realizations reported. Arrows indicate the probabilities that mismatch their phase-randomized counterpart, with the sign symbols representing if they are larger or lower.

It is well known that Gaussian processes have a symmetric ordinal pattern distribution as a result of the distribution of the original amplitude values [36]. For totally uncorrelated data and D=3, the probabilities are clustered into two groups: $p_{\pi_{1}}=p_{\pi_{6}}=1/4$ and $p_{\pi_{2}}=p_{\pi_{3}}=p_{\pi_{4}}=p_{\pi_{5}}=1/8$. We found that such a symmetric clustering is still valid for α<2 and β=0, as can be observed in Figure 3a, which depicts the case of α=0.5 as a representative result of all values of α∈[0.5,2]. The distribution matches the one obtained from the phase-randomized data (Gaussian). The same results are found for other values of the stability exponent and β=0. This inherited property is the reason why the deviation from Gaussianity when β=0 is not captured by the $Z_{pJSD}$ for D=3—see Figure 2a. On the contrary, for a positive skewed distribution, the symmetric clustering property is no longer valid, as observed in Figure 3b. This result is qualitatively representative of other values of β. For D=4, we found quite interesting results for the symmetric case. The ordinal pattern distribution is still symmetrically clustered as its Gaussian counterpart, yet, some probability weights differ, as observed in Figure 3c. The probabilities that differ are marked with an arrow. The mismatch weights are responsible for $Z_{pJSD}$ detecting the symmetric deviation from Gaussianity—see Figure 2b. For the sake of comparison, Figure 3d shows the ordinal distribution for the asymmetric case β=1 (note that the *y* axis is in logarithmic scale for better visualization of the probabilities). The same conclusions as for D=3 can be drawn from this result. For negative skewed cases, β<0, the ordinal pattern distribution is a mirror image of the one obtained for the positive skewed cases. Similar results are found for D=5 and 6.

**Figure 4:**
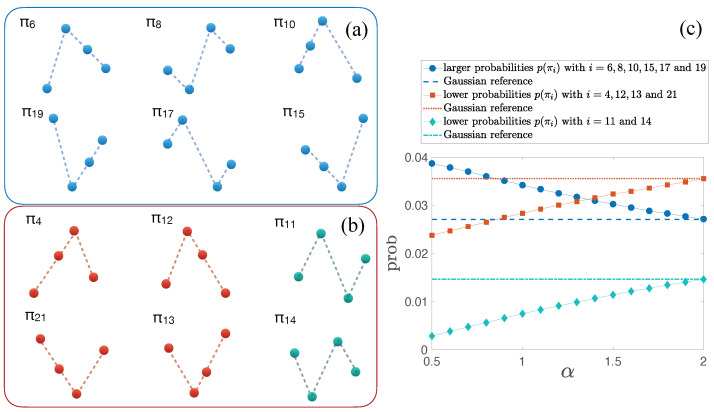
Ordinal patterns whose probability of occurrence is (**a**) larger and (**b**) lower than the ones obtained from phase-randomized data. (**c**) Ordinal pattern probabilities for D=4 that differ from their phase-randomized counterpart for the symmetric case (β=0) as a function of α.

Let us reflect on the symmetric case. From Figure 3c, we observe that for D=4 some patterns are less frequent ($\pi_{4}$, $\pi_{11}$, $\pi_{12}$, $\pi_{13}$, $\pi_{14}$, and $\pi_{21}$), while others are more frequent ($\pi_{6}$, $\pi_{8}$, $\pi_{10}$, $\pi_{15}$, $\pi_{17}$, and $\pi_{19}$) than their phase-randomized counterparts—see arrows in Figure 3c. These patterns are represented in Figure 4a,b, respectively. As the stability exponent α decreases (departures from Gaussianity), the tails of the distribution become heavier, i.e., the probability of large values becomes higher, which is the trigger of having more jumps in the walk. It can straightforwardly be concluded that these jumps are translated to the ordinal patterns depicted in Figure 4a. While the occurrence of these patterns increases with α, other permutations’ frequencies decrease, which are represented in Figure 4b. The evolution of these probabilities with α is shown in Figure 4c. We observe that they gather into three groups. For α=2, all the probabilities match the Gaussian reference—dotted, dashed, and dotted–dashed lines. As α decreases, the six jump-related patterns increase their probability, while the remaining patterns do the opposite. It becomes evident that ordinal patterns decode information about the amplitude when analyzing the walk of non-Gaussian increments.

**Figure 5:**
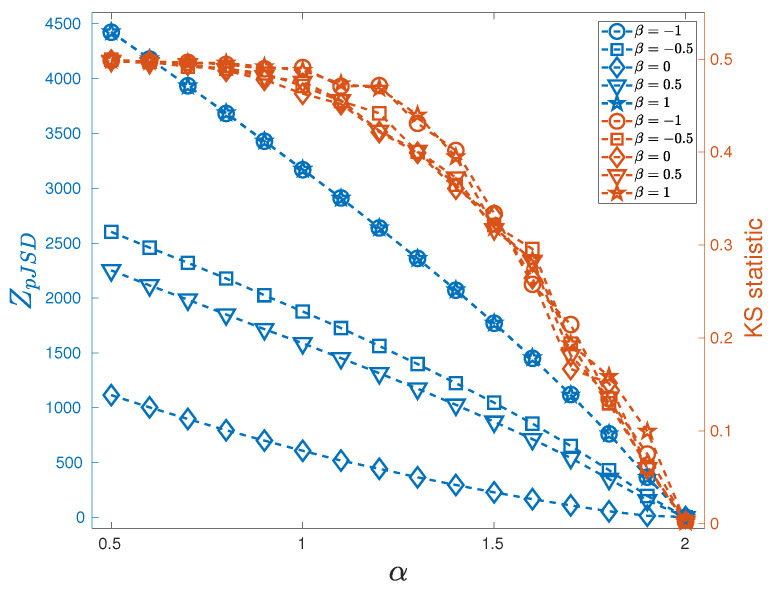
Comparison between $Z_{pJSD}$ (D=4) and the Kolmogorov–Smirnov statistics for sequences following a stable distribution as a function of the parameter α∈[0.5,2] with a step of 0.1 and β=−1,−0.5,0,0.5, and 1. Synthetic sequences of $M=10^{5}$ data points. Average over 100 independent realizations is reported.

To validate the effectiveness of the $Z_{pJSD}$ metric as a measure of non-Gaussianity, we compared it with the well-established Kolmogorov–Smirnov (KS) statistic, a widely used non-parametric test for evaluating if a data sample comes from a given reference probability distribution [37]. Figure 5 summarizes these results. Both metrics show a decreasing trend as α increases. The KS statistic curves are closely clustered, indicating that they do not differentiate between different skewness values. Conversely, $Z_{pJSD}$ exhibits a clear stratification across different β values, indicating better sensitivity to skewness effects. Furthermore, in the heavy-tailed regime (0.5<α<1), the KS statistic saturates, while the $Z_{pJSD}$ continues growing substantially, suggesting it can differentiate stronger deviations from Gaussianity even when KS reaches its limits. Actually, the $Z_{pJSD}$ exhibits a significantly larger numerical range than the KS statistic. This analysis indicates that pJSD is more sensitive in distinguishing deviations from Gaussianity. While the KS statistic provides a global assessment of distributional differences, it does not inherently account for the ordinal structure or local deviations in the amplitude distribution.

In order to further quantify asymmetric deviations from Gaussianity (β≠0), we define a permutation asymmetry metric by computing the average of the sum of differences between symmetrically paired terms as

(6) $$\mathrm{pA}\left(D\right)=\frac{2}{D!}\sum_{i=1}^{D!/2}\left(p_{i}-p_{D!/2-(i-1)}\right).$$

Here, $p_{i}$ represents the *i*-th term of the distribution, and $p_{D!/2-(i-1)}$ represents the symmetrically opposite term in the distribution. In this manner, we can quantify the sign of the amplitude asymmetry of the data, i.e., the sign of β. Additionally, for normalizing this ordinal asymmetry metric, we use the same approach applied to the pJSD, defining a Z-score using the average and the standard deviation of the values resulting from the asymmetry of a set of one hundred independent phase-randomized realizations from the synthetic data:

(7) $$Z_{pA}=\frac{pA-\langle pA_{FFT}\rangle }{\sigma_{pA_{FFT}}}.$$

**Figure 6:**
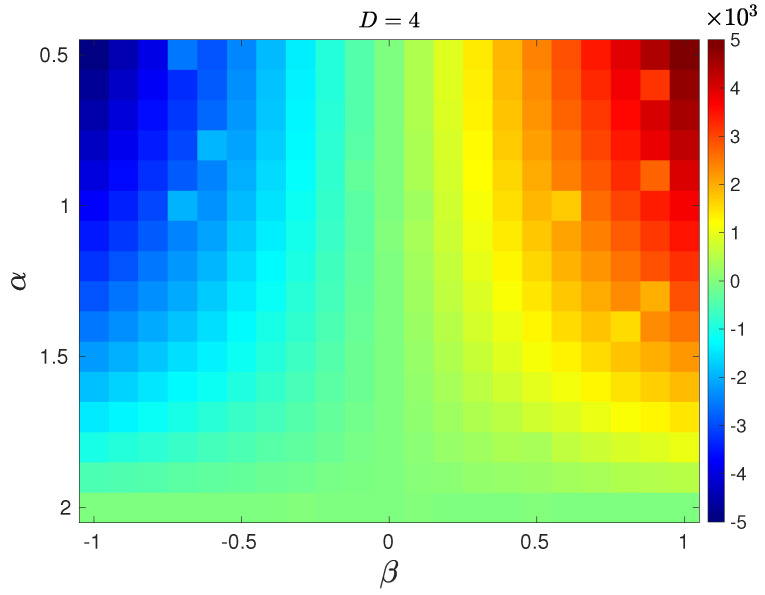
$Z_{pA}$ of the ordinal pattern distribution of the walk from sequences following stable distribution as a function of the parameter β and α. Synthetic sequences of $M=10^{5}$ data points. Average over 100 independent realizations is reported.

Figure 6 depicts the results of this analysis for the data following a stable distribution. We observe that as the value of β moves away from zero, the asymmetry $Z_{pA}$ correctly quantifies the sign of the skewness. Moreover, for β=0, the permutation asymmetry is equal to zero independently of the value on α. Naturally, for α=2, the $Z_{pA}$ is also zero. In this manner, the $Z_{pJSD}$ and the $Z_{pA}$ characterize the entire distribution of the increments of the walk.

## 4. Application to Flight Delays Distributions

The appearance of delays in air transport is both a common occurrence and one of its main problems. Beyond negatively impacting the perception that customers have of this transportation mode, delays have profound implications in the cost-efficiency [38] and safety of the system [39]; they further negatively impact the environment by unnecessarily increasing carbon dioxide emissions [40]. Broadly speaking, these can be caused by random occurrences, such as, e.g., adverse weather phenomena or equipment failures, but also by systemic inefficiencies. We are here going to analyze a large data set of real operations, both in Europe and the US, and show how the proposed metric of distance from Gaussianity can help to discriminate between the two sources.

### 4.1. Analysis of Delay Distributions

As a consequence of the importance of delays in air transport, many studies have tried to provide a deeper and more comprehensive understanding of the factors and dynamics behind flight delays and their propagation, also focusing on their distributions. Note that, were delays of a completely stochastic nature, such a statistical approach would indeed be motivated.

Among the first works to use this approach, Mueller et al. [23] provided a comprehensive characterization of delay distributions at ten major US airports over a 21-day period. They found that departure delays are best modeled using a Poisson distribution, while en-route and arrival delays follow a Normal distribution. Subsequently, Tu et al. [21] found that the departure delay distribution was best modeled using a finite mixture of four normal distributions, exhibiting heavy tails and right skewness, reflecting the different operational and stochastic factors affecting flight delays. The model was trained and validated using United Airlines flight data from Denver International Airport for the years 2000–2001. More recently, Z. Szabó found that the delay distributions in both Europe and the US are well fitted by a non-central Student’s t-distribution [22].

Moving to the analysis of causes, Cao et al. [41] classified all influencing factors into propagation and non-propagation factors (NPFs). To illustrate, bad weather can randomly delay a few operations (what are also called “primary” delays); conversely, those delays may spread to subsequent operations of the same aircraft (i.e., generating “secondary” or “reactionary” delays). Considering the departure delays for Delta Airline, they showed that the ones attributed solely to NPFs adhere to a power law distribution, while the overall flight departure delay distribution follows a shifted power law. On the other hand, Wang and co-workers [20] extended the analysis to multiple operators and divided departure delays from 14 American airlines into the two aforementioned groups; they respectively exhibited a shifted power law or an exponentially truncated shifted power law decay. To further investigate delay distributions, by a comprehensive statistical analysis of flight delays at major UK airports, Mitsokapas et al. [19] identified a power-law decay in large positive delays and an exponential decay in early arrivals.

On this topic, it is further worth considering the work of Wang and coauthors [24], proposing machine learning models to predict the distributions of flight delays, and validating the methods using empirical data from Guangzhou Baiyun International Airport. They examined multiple probability distributions for modeling flight delays, including Beta, Erlang, and Normal distributions. The results suggested that the Normal distribution is better able to capture the stochastic nature of flight delays.

### 4.2. Real Operational Data

We extracted a time series of delays for aircraft arriving at the 50 largest European and US airports, ranked according to the respective number of operations. Data for Europe have been obtained from the EUROCONTROL’s R&D Data Archive, freely accessible at https://www.eurocontrol.int/dashboard/rnd-data-archive (accessed on 24 February 2025), and corresponding to all operations executed throughout March, June, September, and December between the years 2015 and 2019. Note that these four months correspond to a limitation at the source, and do not correspond to a decision of the authors. Data for the US have been obtained from the Reporting Carrier On-Time Performance database of the Bureau of Transportation Statistics, US Department of Transportation, freely accessible at https://www.transtats.bts.gov (accessed on 24 February 2025). In order to obtain comparable results, data have been filtered according to the same dates as in the European set.

**Figure 7:**
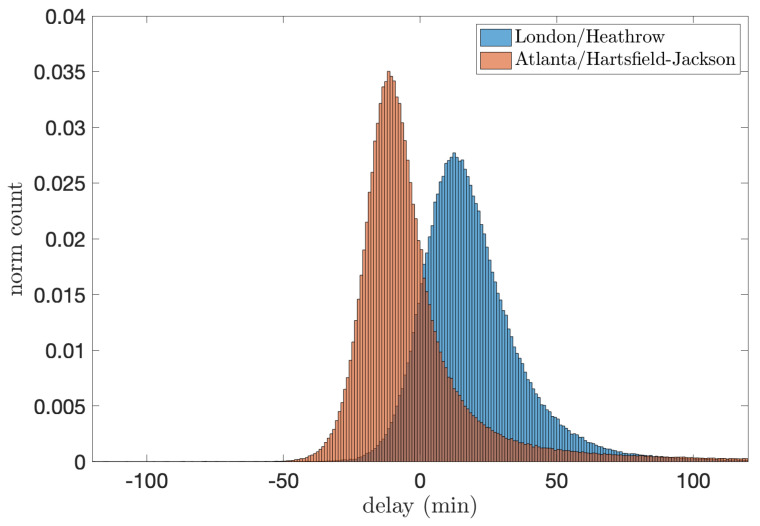
Distribution of the arrival delays in minutes for London/Heathrow and Atlanta/Hartsfield–Jackson, i.e., the biggest airports in Europe and the US, respectively.

In both cases, two delays have been calculated for each flight: the arrival (or landing) delay, defined as the difference between the actual and planned landing times, and the en-route delay, calculated as the increase in delay observed between the take-off and landing. In order to create two individual time series per airport (i.e., one for arrival and one for en-route delays), these values have been concatenated according to the arrival sequence, in which each element is the delay $\delta_{t}$ of a single aircraft. To eliminate any daily and weekly oscillations [42,43] between aircraft, we have shuffled all sequences. In that manner, the distribution of ordinal patterns will only capture the information on the shape of the amplitude distribution. For illustration, Figure 7 shows the distribution of arrival delays for London–Heathrow and Atlanta airports, the biggest airports in Europe and the US, respectively. It can be appreciated that both distributions are asymmetric and positively skewed around their mode.

Following the previous methodology, we here compute the walk of the individual delays $\delta_{t}$ as $D_{i}=\sum_{t=1}^{i}\left(\delta_{t}-\gamma \right)$ with $i\in \{1,2,3,\ldots ,N_{v}\}$ and $N_{v}$, the number of arrivals of each airport. Normally, the parameter γ would represent the mean of the distribution. However, as we aim to characterize the interplay between early (negative delays) and late arrivals (positive delays), the mean does not adequately represent the appropriate reference value; zero, i.e., the operation according to the plan, instead does. Similar to when we want to determine whether a distribution of financial returns is symmetric with respect to zero (i.e., if there are more days with large losses or large gains), we can define a skewness around zero as the reference point. Thus, by setting γ=0, we will be quantifying the exchange between severe delays and early arrivals. For all analyses below, we set D=4 and τ=1.

### 4.3. Results

**Figure 8:**
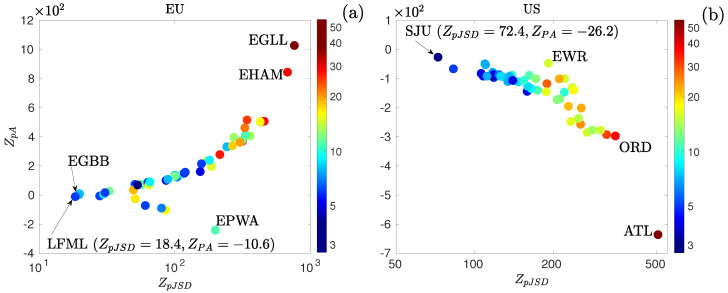
$Z_{pA}$ versus $Z_{pJSD}$ in the semi-logarithmic scale for the distribution of arrival delays at the 50 largest airports of (**a**) Europe and (**b**) the US. The color map indicates the median arrival volume per hour (in logarithmic scale).

Figure 8 reports the asymmetry $Z_{pA}$ as a function of the permutation distance $Z_{pJSD}$, both computed from the ordinal distribution of the walk of the individual arrival delays to their phase-randomized surrogate sequences for (a) EU and (b) US arrival delays. Additionally, the color map indicates the median arrival volume per hour, as a proxy of the size of the airport. Note that similar results are found when using other variables such as the number of arrivals or the number of passengers. Independently of the region, we observe a tendency to draw away from Gaussianity as the arrival volume of the airports increases. To illustrate, in the case of the EU, London–Heathrow (EGLL) airport is the farthest away from it and one of the biggest, together with Amsterdam–Schiphol (EHAM)—see Figure 8a. Similarly, Atlanta–Hartsfield–Jackson (ATL) and Chicago–O’Hare (ORD) are the furthest from Gaussianity for the US, as observed in Figure 8b. For the latter region, we found that all individual delay distributions are negatively skewed, contrary to the European ones, in which only a few are negatively asymmetric, for instance, the Varsovia–Chopin (EPWA) airport. These results indicate that, for the US, there are more early arrivals than severe delays. Note that no distributions can be categorized as Gaussian, considering that the closest to it are the Marsella Provenza (LFML) and Luis Muñoz Marín (SJU) airports in the EU and US, with $Z_{pJSD}=18.4$ and 72.4 and $Z_{pA}=-10.6$ and −26.2, respectively—see Figure 8.

**Figure 9:**
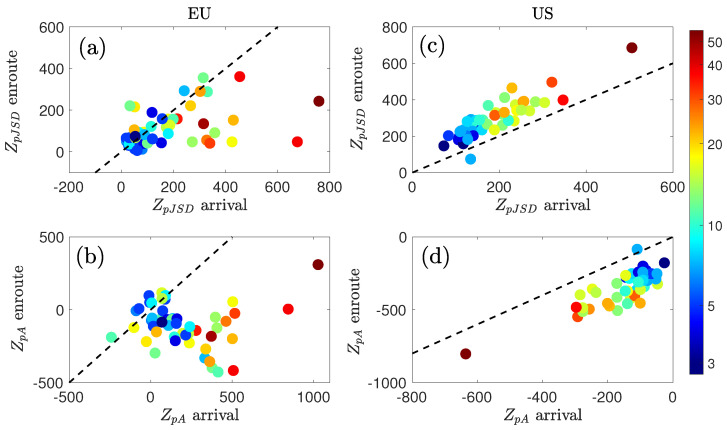
$Z_{pJSD}$ and $Z_{pA}$ for the distributions of en-route versus arrival delays for (**a**,**b**) EU and (**c**,**d**) US, respectively. Black dashed line indicates the identity line. The color map indicates the median volume per hour (in logarithmic scale).

Figure 9 shows a scatterplot of the Z-scores for en-route delays, as a function of those for arrival delays. For the EU (left panels), a deviation from Gaussianity similar to the previous one is observed for both distributions. Conversely, in the case of the US (right panels), en-route distributions are more distant from the Gaussian assumption.

In order to understand these results, it is necessary to recall how unexpected events are managed in both systems. In Europe, delays on the ground are prioritized; for instance, when the capacity of the destination airport is reduced, flights are held on the ground until there is a high certainty about their arrival—this is done through “slots”, or authorizations to departure within a given time window [44]. As a consequence, once a flight has departed, it should not expect delays en-route, and most of the time, the trip ends up being shorted, through directs and other tactical decisions. On the contrary, the US system relies more strongly on delays while en-route, except for some circumstances handled through the Ground Delay Program [45]. Note that no approach is inherently better: while the European one avoids en-route delays and their associated environmental impact, the US system is more flexible and can handle disruptions on shorter time scales.

Going back to the results presented in Figure 9, it can be appreciated that both Z-scores are highly correlated between en-route and arrival in the US case. In other words, whenever a disruption affects the system, this responds with a similar behavior in the two conditions, thus in a way aligned with what was previously described. The EU system is more complex, with a less clear correlation and more heterogeneous dynamics. On the one hand, $Z_{pA}$ is usually negative: the system tends to favor negative delays while en-route, i.e., aircraft can recover part of their departure delay and not add additional ones. On the other hand, $Z_{pJSD}$ in the en-route phase are positive and weakly related to the ones in the arrival phase. If a deviation from Gaussianity is interpreted as the appearance of systemic (as opposed to random) disruptions, this result implies that en-route abnormal delays are the result of a disrupted global state.

**Figure 10:**
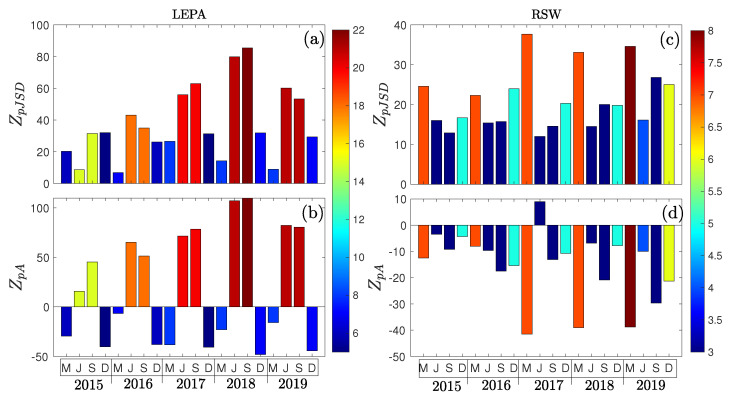
Monthly evolution of the Z-Scores $Z_{pJSD}$ and $Z_{pA}$ for LEPA (**a**,**b**) and RSW (**c**,**d**), respectively. Color maps indicate the median arrival volume per hour.

We further study how deviations from Gaussianity evolve through time. For this, we select two airports for each region of study that present a considerable seasonal variability in traffic and demand. In EU, Mallorca is one of the main tourist destinations in the Mediterranean, attracting over 10 million visitors annually. Its airport, Son Sant Joan (LEPA), is the third most important airport in Spain in terms of passenger volume, which makes it a good example to study the monthly change in the distribution of delays. For the US, the Southwest Florida International Airport (RSW) is located in a region popular for seasonal visitors, including “snowbirds”(a colloquial term for people, often retirees, who migrate seasonally from colder northern regions to warmer southern areas, such as Florida, during the winter months). This airport experiences a steep drop in traffic when the peak winter vacation season ends. Figure 10 reports the results for the monthly analysis of arrival delays at these airports. On the one hand, LEPA shows a deviation from Gaussianity perfectly aligned with its size. The permutation asymmetry changes its sign between seasons, being negative for winter months and positive for summer ones—see Figure 10b—meaning that there exists a strong correlation between severe delays and the saturation of the airport. On the other hand, even though RSW also shows a correlation between the arrival volume and its distance to its Gaussian reference, its distribution is always negatively skewed (except for June 2017), following the general tendency for airports located in the US—compare with Figure 8b.

**Figure 11:**
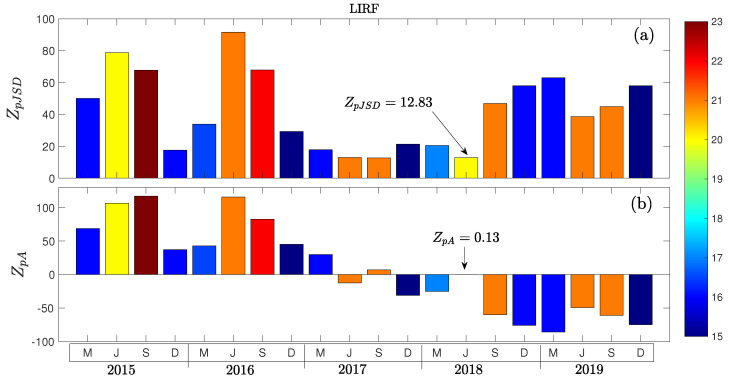
Monthly evolution of the Z-Scores (**a**) $Z_{pJSD}$ and (**b**) $Z_{pA}$ for Leonardo da Vinci-Fiumicino Airport (LIRF). Color maps indicate the median arrival volume per hour.

Finally, we study the busiest airport in Italy, serving Rome, the Leonardo da Vinci-Fiumicino Airport (LIRF); this airport has been selected for displaying very interesting and unusual dynamics. As shown in Figure 11a, deviations from Gaussianity are quite heterogeneous and do not scale with arrival volume, as the general trend observed for the concatenated data (Figure 8a). Even more interesting is the transition found in September of 2017, after which the asymmetry $Z_{pA}$ goes from being positive to negative, implying that the airport went from having more severe delayed arrivals to early ones. Moreover, for June of 2018, we found that the distribution corresponds to a Non-Gaussian symmetric one. While it is difficult to provide a complete explanation, several factors may have contributed to this shift in dynamics. Specifically, starting from the year 2017, the airport of Rome Fiumicino has experienced a substantial decrease in traffic—mostly due to the problems experienced by the main airline operating there, i.e., Alitalia, currently ITA Airways [46]. This, along with more efficient runway operations, a more spread distributions of operations throughout the day and away from peak hours, and the more efficient management of flights as part of the Local Single Sky ImPlementation plan [47], resulted in a substantial decrease in delays.

## 5. Conclusions

In this study, we have introduced a simple data-driven approach to quantify deviations from Gaussianity in time series. By computing the Jensen–Shannon distance permutation between synthetic data sampled from stable distributions and their phase-randomized surrogates, we provide a statistical framework that effectively captures the characteristics of heavy tails and skewness. It is noteworthy that, rather than incorporating information about the amplitude distribution when building the ordinal patterns, the present approach demonstrates that quantifying the ordinal dissimilarity between cumulative integration (walk) of the sequences under study and the Gaussian reference is sufficient to explore non-Gaussian features.

Our findings reveal several interesting facts about delay distributions across airports and their relationship with traffic. These distributions at major European and US airports exhibit significant departures from normality, with US airports predominantly showing negative skewness, indicating a preference for early arrivals over severe delays. In contrast, European airports display more heterogeneous skewness patterns. These results highlight fundamental differences in air traffic management strategies between the two regions. Furthermore, higher traffic volumes correlate with deviations from Gaussianity, particularly in seasonal hubs such as Mallorca and Southwest Florida. This implies that congestion plays a crucial role in shaping the statistical properties of delays. Our study of Rome Fiumicino Airport also revealed a structural transition in its delay dynamics, likely influenced by operational and airline management changes.

Our approach enhances the understanding of non-Gaussian properties in delay distributions. While such knowledge cannot trivially be translated to the improvement of operational procedures, the analyses proposed here could be used to monitor the delay dynamics over time, especially when changes are introduced in the system—as seen, for instance, in the case of Rome Fiumicino. Consequently, this approach could be part of solutions designed to monitor the efficiency of airports, a topic of major relevance in the literature [48,49,50], yet for which good solutions are still not available [51]. In addition to providing this descriptive insight, this approach offers a computationally efficient and model-free tool for detecting systemic deviations from expected (Gaussian) behavior. These deviations may indicate congestion or systemic inefficiencies in an airport or airspace region during real-time monitoring. This ordinal dissimilarity allows for the evaluation of different air traffic management strategies across airports. Moreover, identifying where Gaussian assumptions fail contributes to the development of more accurate forecasting models, as Gaussianity is assumed in many of them, especially when not enough data are available for a complete characterization [52,53,54].

Beyond the characterization of flight delays, our methodology provides a general approach for identifying non-Gaussian signatures in complex systems. It offers a computationally fast, simple, and model-free technique that can be applied to other fields where deviations from normality play a critical role, such as financial time series [55] and physiological data [56].

## Data Availability

No new data were created or analyzed in this study. The original data analyzed in the study are openly available at https://www.eurocontrol.int/dashboard/rnd-data-archive and https://www.transtats.bts.gov (accessed on 24 February 2025).
